# Stomachic Bitters, Tonics, and Aperients

**Published:** 1855-02

**Authors:** G. Budd


STOMACHIC BITTERS, TONICS, AND APERIENTS.—

*Quinine* strengthens digestion, especially when the stomach has been injured by drinking, or in persons exhausted by overwork. It does harm in organic disease, with a furred tongue, and when the urine throws down lithic acid, or lithate of ammonia. Give quinine half an hour or an hour before meals.

*Columba.*—This is a sedative as well as a tonic.

*Gentian and Chiretta* stimulate the liver, as well as act as tonics on the stomach; whereas, quinine and quassia sometimes check the secretions of the liver, and are therefore not adapted to cases where tne secretion of bile is defective.

*Iron.*—The citrate, or ammonia-citrate, mixed with a little bicarbonate of soda or potash, have the same effect as Griffith's mixture. Iron medicines may be given at meal-times.

*Castor-Oil* acts very mildly on the large intestines, and is therefore useful in convalescence from typhoid fever, in dysentery, and whenever we wish not to act too much on the large intestines.

*Senna* acts chiefly on the small intestines, and increases the secretion from the mucous membrane, and also that of the liver. It is, therefore, one of the best aperients in bilious affections.

*Aloes and Colocynth* act chiefly on the large bowels, and are, therefore, the best purgatives in disorders of the stomach.—*Dr. G. Budd.*

